# Ibrutinib and venetoclax target distinct subpopulations of CLL cells: implication for residual disease eradication

**DOI:** 10.1038/s41408-021-00429-z

**Published:** 2021-02-18

**Authors:** Pin Lu, Shengchun Wang, Carrie A. Franzen, Girish Venkataraman, Rebecca McClure, Lei Li, Wenjun Wu, Nifang Niu, Madina Sukhanova, Jianming Pei, Donald A. Baldwin, Reza Nejati, Mariusz A. Wasik, Nadia Khan, Yifan Tu, Juehua Gao, Yihua Chen, Shuo Ma, Richard A. Larson, Y. Lynn Wang

**Affiliations:** 1 Fox Chase Cancer Center, Philadelphia, PA USA; 2 Guangdong Medical University, Dongguan, China; 3 University of Chicago Medicine, Chicago, IL USA; 4 Health Sciences North Research Institute, Sudbury, ON Canada; 5 St. Louis University, St Louis, MO USA; 6 Northwestern University Feinberg School of Medicine, Chicago, IL USA

**Keywords:** Translational research, Haematological diseases

## Abstract

Ibrutinib inhibits Bruton tyrosine kinase while venetoclax is a specific inhibitor of the anti-apoptotic protein BCL2. Both drugs are highly effective as monotherapy against chronic lymphocytic leukemia (CLL), and clinical trials using the combination therapy have produced remarkable results in terms of rate of complete remission and frequency of undetectable minimal residual disease. However, the laboratory rationale behind the success of the drug combination is still lacking. A better understanding of how these two drugs synergize would eventually help develop other rational combination strategies. Using an ex vivo model that promotes CLL proliferation, we show that modeled ibrutinib proliferative responses, but not viability responses, correlate well with patients’ actual clinical responses. Importantly, we demonstrate for the first time that ibrutinib and venetoclax act on distinct CLL subpopulations that have different proliferative capacities. While the dividing subpopulation of CLL responds to ibrutinib, the resting subpopulation preferentially responds to venetoclax. The combination of these targeted therapies effectively reduced both the resting and dividing subpopulations in most cases. Our laboratory findings help explain several clinical observations and contribute to the understanding of tumor dynamics. Additionally, our proliferation model may be used to identify novel drug combinations with the potential of eradicating residual disease.

## Introduction

Chronic lymphocytic leukemia (CLL) is the most common leukemia/lymphoma among older adults. It is a chronic lymphoid malignancy with an accumulation of monoclonal mature B-cells in peripheral blood (PB), bone marrow (BM), and secondary lymphoid tissues, such as spleen and lymph nodes (LN)^1,2^.

Molecularly targeted therapies have produced remarkable therapeutic benefits in CLL. Ibrutinib is a small molecule inhibitor of Bruton tyrosine kinase (BTK), which plays a key role in the B-cell receptor (BCR) signaling. Venetoclax, on the other hand, is a highly selective inhibitor of the anti-apoptotic protein BCL2. Both drugs are highly effective as monotherapy against CLL^3–6^, and clinical trials of the combination therapy are ongoing. An interesting clinical observation is that sensitivity of tumor cells to these two drugs depends on anatomic compartments. In general, many patients receiving ibrutinib experience rapid reduction in lymphadenopathy while patients exposed to venetoclax frequently have tumor lysis. These observations suggest lymph node-resident CLL cells are more sensitive to the action of ibrutinib, while circulating CLL cells in the PB are more sensitive to the action of venetoclax. When these two drugs are used in combination to treat relapsed/refractory^7^ or previously untreated CLL patients^8^, the rates of complete response were significantly higher than those reported for either single drug alone. Importantly, undetectable BM minimal residual disease (uMRD), which is rarely observed with ibrutinib alone, has been achieved in patients, and the rate of BM uMRD continues to increase over time.

However, the reasons behind these observed differential compartmental responses and how it is related to the tumor cellular behavior is largely unknown. A better understanding of how these two drugs synergize would eventually help develop other rational combination strategies aimed at eliminating CLL cells from all anatomic quarters.

Concerning the cellular behaviors of CLL, it has been historically thought that CLL is a disease with defects in apoptosis since tumor cells isolated from the peripheral circulation are dormant and non-proliferating. In the last several years, it has been increasingly recognized that cell proliferation also plays an important role in the CLL pathogenesis^9^. Using heavy water tracing of the leukemia cells in patients, it was shown that up to 1% of circulating CLL cells (~1 × 10^9^ cells) are newly generated each day from the BM and LN^10^. At these tissue sites, tumor cells, with the support of the tumor microenvironment, are actively proliferating, particularly in histological tissue areas identified as proliferation centers^11,12^. Proliferation centers are the major histological sites for CLL pathogenesis, progression, and transformation^13^. Larger centers with high proliferation index predict aggressive clinical behavior and poorer patient survival^14^.

Due to easy access, primary tumor cells isolated from the PB of CLL patients are most widely utilized for the purpose of drug testing. The ex vivo approach is cost-effective and is achievable in a short time frame, but it does not reproduce tumor in vivo behaviors. Although CLL has become known as a disease with increased proliferation, isolated PB CLL cells are non-proliferative and undergo spontaneous apoptosis in 2–7 days ex vivo^15^. Drug testing normally evaluates if a particular agent induces additional apoptosis of isolated CLL cells, but frequently such agents turn out to be ineffective in humans. On the contrary, as a successful drug, ibrutinib generated only an average of 10% additional cell killing in a cohort of patients when tested on PB CLL cells^16^. These observations suggest that apoptosis testing with PB is highly unreliable and clinically irrelevant.

With the increasing understanding of the tumor microenvironment, attempts have been made to recreate the tissue tumor niche in vitro. In the LN microenvironment, CLL tumor cells interact with T cells, dendritic cells, and various other types of stromal components^17–19^. It has been shown that the tumor microenvironment protects CLL cells from spontaneous and drug-induced apoptosis^20–22^. Protection by the microenvironment may also be responsible for the persistence of minimal residual disease (MRD), and shorter progression-free and overall survival in treated patients^23^. In this study, using an ex vivo model that is capable of promoting robust CLL proliferation, we investigated how CLL cells respond differently to ibrutinib, venetoclax, and the combination. Our results revealed that ibrutinib and venetoclax act on distinct subpopulations of CLL cells, and help explain several clinical observations related to the use of these highly effective drugs.

## Materials/subjects and methods

### Reagents and antibodies

For CLL cell isolation and ex vivo co-culture, RosetteSep™ Human B Cell Enrichment Cocktail was purchased from Stemcell Technologies (Vancouver, Canada), Dynabeads™CD3 from Thermo Fisher Scientific (Waltham, MA), CpG (ODN2006) from InvivoGen (San Diego, CA), Recombinant human Interleukin-15 (IL-15) from Gemini Bio-Products(West Sacramento, CA), and CellTrace™ Violet Cell Proliferation Kit from Thermo Fisher Scientific (Waltham, MA). For flow cytometry analysis, FITC anti-human CD5 (Cat# 300606), PE anti-human CD19 (Cat# 302254), APC anti-human CD3 (Cat# 300458), APC anti-human CXCR4 (Cat# 306510) and Alexa Fluor 647 anti-human Ki67 (Cat# 652408) antibodies were purchased from Biolegend (San Diego, CA). Propidium iodide from Sigma (St. Louis, MO). For drug treatment, ibrutinib and venetoclax were purchased from Selleckcham, (Houston, TX).

### Clinical samples and CLL cell isolation

CLL patient PB samples were collected after informed consent according to the Declaration of Helsinki. The study was approved by the Institutional Review Board of the University of Chicago. Clinical characteristics of the patients were shown in Table 1. CLL cells were isolated by negative selection using Rosettesep^TM^ human B cell enrichment cocktail following the manufacturers’ instructions as previously described^15^. After isolation, CLL cell purity was assessed using flow cytometry and was >95% CD19+/CD5+ in all cases. Cell viability was assessed with MUSE Count & Viability Kit and was ≥90% in all cases.

**Table 1:** Patient clinical and pathological characteristics.

Pt ID	Age	Sex	Therapy^a^	Rai stage	ZAP-70	IGHV	Cytogenetic abnormalities	Ibrutinib resistance	BTK mutation	Proliferating fraction in untreated control
CCLL011	42	M	T	1	Pos	UM	Trisomy 12	No	No	2.7
CCLL015	56	F	T	1	Pos	UM	Trisomy 12	No	No	36.2
CCLL094	62	F	NT	1	Neg	M	del(13q14.3)	No	No	21.8
CCLL189	60	M	T	4	−	UM	del(13q14.3), del(11q22.3)	No	No	60.8
CCLL248	67	F	NT	3	Neg	M	del(13q14.3)	No	No	19.3
CCLL292	77	F	T	4	Neg	UM	del(13q14.3), del(17p)	No	No	31.3
CCLL306	68	M	T	3	Pos	UM	Normal	No	No	20.0
CCLL320	65	F	NT	4	N/A	UM	Normal	No	No	15.9
FCLL003	85	F	T	N/A	N/A	UM	Normal	No	No	62.5
FCLL008	65	F	T	3	N/A	UM	Complex, del(11q22.3), del(13q14.3)	No	No	66.5
FCLL009	87	F	T	1	N/A	UM	Complex, del(11q22.3), del(13q14.3)	No	No	89.7
FCLL012	61	M	NT	0	N/A	N/A	del(11q22.3), del(13q14.3)	No	No	44.4
FCLL016	56	M	NT	1	N/A	M	del(13q14.3)	No	No	87.0
FCLL018	65	M	NT	0	N/A	N/A	del(13q)	No	No	3.1
FCLL020	74	M	NT	1	Neg	M	del(13q14.3)	No	No	4.8
SCLL025	53	M	NT	2	Pos	UM	Normal	No	No	18.5
SCLL034	67	M	NT	0	Pos	M	Normal	No	No	55.6
UCLL040	77	M	T	0	Pos	N/A	del(11q22.3), trisomy 12	No	No	85.7
UCLL046	36	M	NT	1	N/A	M	Normal	No	No	70.8
UCLL047	64	M	NT	0	Neg	UM	Trisomy 12	No	No	83.1
UCLL053	45	M	NT	N/A	Ind	M	del(13q14.3)	No	No	51.5
UCLL057	88	F	T	3	N/A	UM	del(13q14.3)	No	No	4.9
UCLL058	40	M	T	2	Ind	UM	del(13q14.3)	No	No	6.2
NCLL018	47	M	T	2	Pos	UM	del(11q22.3), del(13q14.3)	**Yes**	***BTK***^***C481S***^	82.9
NCLL021	72	M	T	0	N/A	UM	Trisomy 12	**Yes**	No	92.1
UCLL011	57	F	T	N/A	Pos	UM	del(17p)	**Yes**	***BTK***^***C481S***^	65.8
UCLL017	68	M	T	N/A	Pos	UM	del(17p)	**Yes**	No	15.3
UCLL034	50	M	T	4	Pos	UM	del(13q14.3)	**Yes**	***BTK***^***C481Y***^	85.7
UCLL035	64	M	T	2	Pos	UM	del(11q22.3), del(13q14.3)	**Yes**	***BTK***^***C481S/Y***^	87.9
UCLL041	59	M	T	N/A	Neg	UM	del(17p)	**Yes**	***BTK***^***C481F***^	43.1
UCLL052	56	M	T	N/A	N/A	N/A	del(17p)	**Yes**	***BTK***^***C481S***^	87.3

*N/A* information not available, *Ind* indeterminant, *T* treated, *NT* not treated.

^a^Treatment status at the time of sample collection.

### Generation and maintenance of BM fibroblast (BMF) cell line

The BMF cell line was generated from a long-term culture of the BM cells from a CLL patient without further manipulation. BMF is maintained in RPMI-1640 with 10% fetal bovine serum under standard conditions (5% CO_2_, 37 °C). Further characterization indicates that it is a normal human fibroblast exhibiting monolayer growth and contact inhibition. The cell line is positive for BM mesenchymal cell marker CD44, CD29, and CD71^24^. Conventional cytogenetic analysis revealed a karyotype of 45, X, -Y, which is consistent with the profile of the patient (73 year-old male with the loss of Y chromosome, a phenomenon frequently noted in elder males).

### Pre-culture of CLL cells with BMF, T-cell depletion and CFSE labeling

BMF were trypsinized and seeded onto 12-well plates (2 × 10^5^ cells/2 mL/well) to reach 70–90% confluence on the next day. CLL cells were then added to the BMF monolayer in RPMI-1640 media containing 20% FBS, 50 U/mL penicillin, 50 mg/mL streptomycin and 2 mmol/L of l-glutamine (Mediatech. VA). Plating ratio of CLL to stromal cells fall in the range of 2:1–20:1 depending on the cell number availability in the CLL samples. After 72 h of co-culture, CLL cells were collected by gently pipetting, washed and resuspended in 1 mL media. The residual T cells were removed by Dynabeads®CD3 kit according to manufacturer’s protocol. In brief, CD3 Dynadeads were added at 50 µL per mL cell suspension, and rotated at 4 °C for 30 min. The bead-bound CD3+ T cells were separated from cell suspension by EasySep™ Magnet.

After CD3 depletion, CLL cells were labeled with violet-CFSE using CellTrace™Violet Cell Proliferation Kit according to manufacturer’s protocol. Briefly, 10 µL of 5 mM CFSE in DMSO was added into 10 mL pre-warmed PBS for each labeling. CLL cells were washed twice with PBS and re-suspended in 10 mL CFSE-PBS solution. After a 20-min of incubation at 37 °C, cells were spun down, washed once and kept in RPMI-1640 media with 20% FBS for the next step.

### Cell stimulation and drug treatment

Cell stimulation and drug testing were conducted in 24-well plates with BMF monolayer. For activation of CFSE-labeled CLL cells, CpG (2 µg/mL) and IL-15 (10 ng/mL) were added into each well except the unstimulated control. For drug treatment, ibrutinib or venetoclax, at clinically achievable concentrations, was added to the co-culture 24 h after CpG/IL-15 addition. For the control well, an equal volume of DMSO was added. At day 7 of the stimulation and drug treatment, 400 µL of cultured CLL cells were collected from each well for flow cytometric analysis.

### Cell survival and proliferation analyses

Cell survival and proliferation were determined by flow cytometry using LSR2 flow cytometer (BD Biosciences). Briefly, CLL cells collected at day 7 of drug treatment were stained with FITC-anti-CD5, PE-anti-CD19, APC-anti-CD3 antibodies, and PI solution was added after the antibody staining and before the flow cytometry analysis. Flow acquisition was conducted for the fixed time duration (60 s). Data were analyzed using FlowJo software (Version 10; TreeStar). Live CLL cell number was calculated by counting CD19^+^/CD5^+^ and PI^−^ events in comparison to the DMSO control. For CLL proliferation, the percentage of cells distributed in the dividing phases of the CFSE profiles is automatically calculated by the FlowJo software. For Ki67 staining, CLL cells were first stained with FITC-anti-CD19 antibody, fixed and the permeabilized prior to Alexa Fluor 647 anti-human Ki67 antibody staining.

### Statistical analysis

Statistical analysis of the data was performed using Graphpad Prism 8 software (GraphPad, La Jolla, CA, USA). Two group comparisons were conducted using non-parametric paired *t*-test selected by the Prism 8 software since data points are not normally distributed. One-way ANOVA analysis was applied for comparison among multiple groups and *F*-ratios are shown when they are indicated. *P*-values of 0.05 are considered statistically significant.

## Results

### CLL cells actively divide for a long-term in a co-culture model

**Fig. 1:**
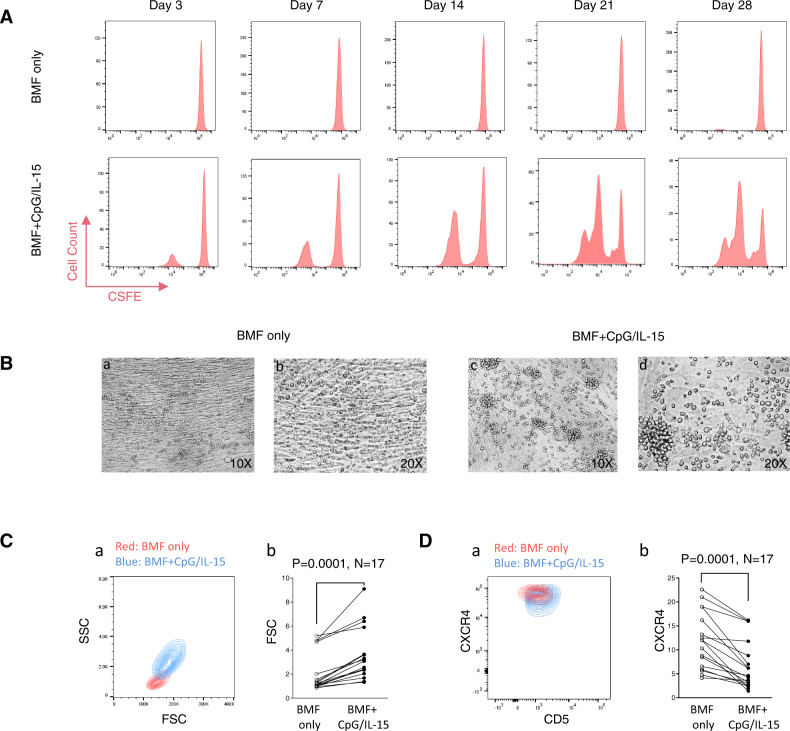
CLL cells actively divide for a long term in an ex vivo model. Peripheral blood CLL cells from a patient were co-cultured with either BMF alone or with BMF+CpG/IL-15 stimulation. **A** Cell proliferation analyzed by flow cytometry. CFSE-labeled CLL cells were cultured with either BMF alone (upper) or with CpG (2 µg/mL) plus IL-15 (10 ng/mL) (lower). Duration of culture were indicated. **B** Light microscopy of CLL cells at Day 7 of the co-culture. CLL cells cultured with BMF alone are single small round cells laying on top of the BMF monolayer (a and b), while CLL cells with CpG/IL-15 stimulation appear larger and clustered (c and d). Microscopic magnifications are indicated. **C** Cell size was determined by forward scatter (FSC) on flow cytometry at Day 7 of the co-culture. SSC/FSC contour map between the two culture conditions is shown for one case (a) and for 17 cases (b). **D** Cell surface CXCR4 was measured by flow cytometry at Day 7 of the co-culture. CXCR4/CD5 contour map between the two culture conditions is shown for one case (a) and for 17 cases (b).

CLL cells isolated from the PB normally survive 2–7 days in vitro without proliferation^15^. Previously, we have constructed a NKTert cell line-CLL co-culture model that supports tumor cell proliferation as evidenced by the appearance of a BrdU^+^ cell population^25–28^. However, BrdU incorporation merely measures DNA synthesis, one step of several in the cell division process. In order to visualize distinct generations of cell division, the model was further improved with a fibroblast cell line (BMF) established from the BM of a CLL patient and with added stimulants, CpG oligonucleotides and IL-15^22,29,30^. In this model, CLL cells, when co-cultured with BMF alone, were maintained alive but showed no signs of cell division as assayed by CFSE staining (Fig. 1A, top panels). In contrast, when CLL cells were co-cultured with BMF plus CpG/IL-15 stimulation, several generations of daughter cells appeared. Cell division started on day 3 and cells continued to proliferate until day 28 (Fig. 1A, compare top vs. bottom panels). The culture, in some cases, survived and proliferated for 10 weeks before cell death ensued.

Under light microscopy, stimulated cells appeared larger and formed clusters (Fig. 1B, compare a with c at 10×, b with d at 20×). In addition, CpG/IL15-stimulated cells, assayed by flow cytometry, were indeed larger compared to cells co-cultured with BMF alone. Figure 1C–a shows change in cell size of a typical case and Fig. 1C–b shows the aggregate data of 17 cases with or without the stimulation. The stimulated cells also expressed lower levels of surface CXCR4 as shown in Fig. 1D–a, b. Downregulations of CXCR4 has been associated with CLL cells that are more proliferative^22,31^. Taken together, data from CFSE labeling, light microscopy, cell size, and CXCR4 expression demonstrate that CLL cells are actively proliferating and dividing in our co-culture model. Although the actual lymph node architecture is far more complex, with its ability to promote long-term cell proliferation, the model recapitulates at least one of the most salient features observed histologically in the “proliferation centers” of the LN. We thus refer this model as the CLL proliferation model.

### Only the dividing subpopulation of CLL responds to ibrutinib, which is largely consistent with patients’ actual clinical response

**Fig. 2:**
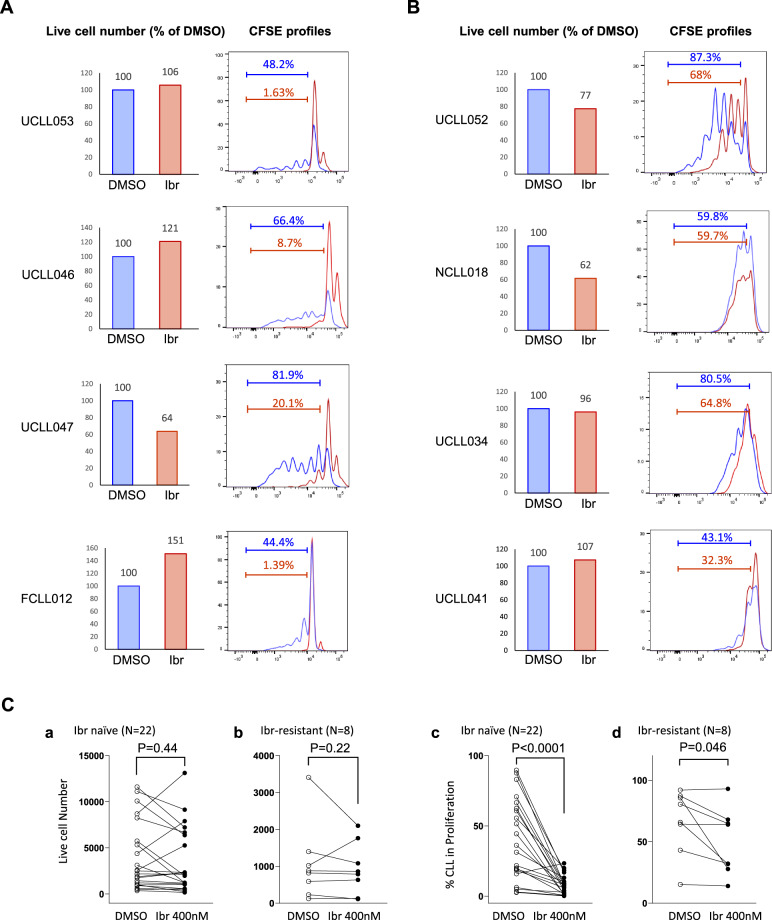
Only the dividing subpopulation of CLL responds to ibrutinib, which is largely consistent with patients’ actual clinical response. CFSE-labeled CLL cells were cultured with BMF+CpG/IL-15 in the presence of DMSO or 400 nM of ibrutinib (Ibr). All analyses shown were conducted at day 7 of the co-culture by flow cytometry. **A** Live cell numbers (bar graphs) and CFSE profiles of 4 ibrutinib naïve/sensitive cases. Live cell numbers were normalize to the DMSO control (100%). Percentage of cells distributed in the dividing phases of the CFSE profiles is automatically calculated by the FlowJo software. **B** Live cell numbers and CFSE profiles of 4 ibrutinib resistant cases. **C** Aggregate results of cell viability and % of dividing CLL cells are shown for ibr naïve patients (a and c) as well as ibr resistant patients (b and d).

Previously, using a less advanced co-culture model, we demonstrated that ibrutinib inhibits the appearance of BrdU-positive CLL cells^25–27,32^. With the current improved CLL proliferation model and optimized conditions including type of stromal cells and time points (data shown in a separate manuscript), we are able to investigate how ibrutinib affects cell division, which has not been studied before in the absence of a model tool. We tested the effects of the drug on cell division as well as on cell viability in samples taken from 22 ibrutinib-naïve patients and eight ibrutinib-resistant patients (Table 1). Only a fixed concentration of 400 nM was used to preserve the clinical samples for multiple assays. The concentration was selected based on the *C*_max_ (420 nM) derived from the published human pharmacokinetic studies^33–35^. Figure 2A shows four cases of ibrutinib-naïve patients. In case UCLL053, after 7-day of culture, cell viability was essentially not changed from the vehicle control **(**Fig. 2A left panel. 100% vs. 106%). In contrast, the CFSE profile shows that generations of dividing cells were significantly scaled down from 48.2% to 1.63% with ibrutinib treatment (compare blue vs red trace). Study of additional cases revealed that ibrutinib treatment caused variable changes in cell viability—minimal changes in UCLL053, decrease in UCLL047, and even increases in UCLL046 and FCLL012 (see “Discussion” section). In comparison to viability, ibrutinib caused a significant and consistent suppression of cell division in all four cases (also see aggregate data later in Fig. 2C).

We also studied the behaviors of cells harvested from ibrutinib-resistant patients. Six of the eight ibrutinib-resistant patients carried the BTK C481 mutation at the time of disease relapse^25,36^ (Table 1). As shown in Fig. 2B, in UCLL052, cell viability was reduced from 100 to 77% comparing DMSO vs. ibrutinib-treated cells. CFSE profile, however, shows that ibrutinib decreased cell proliferation from 87 to 68%. Notably, cell division is much less inhibited in this ibrutinib-resistant case comparing to ibrutinib-naïve patients. Similar observations were also made in other cases where suppression of cell proliferation occurred to a much lesser degree compared to ibrutinib-sensitive cases.

The aggregate data for all 22 ibrutinib-naïve and 8 ibrutinib-resistant patients demonstrate, despite some patient-to-patient variation, lack of a significant inhibition of ibrutinib on cell viability, in both ibrutinib-naïve and ibrutinib-resistant patient cohorts (Fig. 2C–a, b, *p* = 0.44 and 0.22, respectively). This finding is consistent with previous data by us and other groups showing ibrutinib does not induce direct cell death^25,26,32,37^. In comparison, ibrutinib markedly reduced proliferation in cells taken from ibrutinib-naïve patients (Fig. 2C–c, *p* < 0.0001). In ibrutinib-resistant cases, such inhibition occurred at a lower degree and at a lower frequency, so that when the resistant patients were analyzed as a cohort, the inhibition was not as significant (Fig. 2C–d, *p* = 0.046). Overall, these data demonstrate cell division responses to ibrutinib are highly correlated with patients’ *actual* clinical responses to ibrutinib while the conventional cell viability responses are not. Thus, the validity of ibrutinib response readouts by the CLL proliferation model are well supported by patients’ clinical response data.

### The resting subpopulation of CLL, instead of the dividing one, preferentially responds to venetoclax

**Fig. 3:**
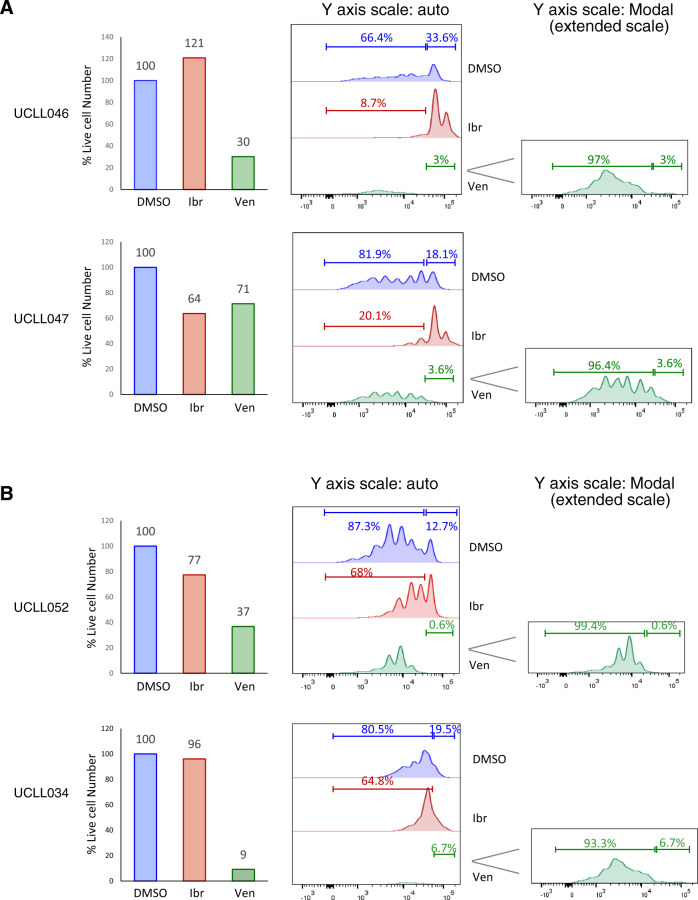
The resting subpopulation of CLL, as opposed to the dividing one, preferentially responds to venetoclax. CFSE-labeled CLL cells were cultured with BMF+CpG/IL-15 in the presence of DMSO, 400 nM of ibr or 200 nM of venetoclax (ven). All analyses shown were conducted at day 7 of the co-culture by flow cytometry. **A** Live cell numbers (bar graphs) and CFSE profiles of two ibrutinib naïve/sensitive cases. Live cell numbers were normalize to the DMSO control (100%). Percentage of cells distributed in the dividing phases of the CFSE profiles is automatically calculated by the FlowJo software. *Y* axis scale-auto displays events on the same scale for all treatment conditions, and *Y* axis scale-Modal displays events on an extended scale to show low peaks under the condition of ventoclax treatment. **B** Live cell numbers and CFSE profiles of two ibrutinib resistant cases.

With the CLL proliferation model, we also evaluated how the BCL2 inhibitor venetoclax works. As shown in Fig. 3A, in an ibrutinib-naïve case, UCLL046, venetoclax, as expected, markedly decreased cell viability from 100 to 30%, at a clinically achievable concentration of 200 nM (Fig. 3A, top left panel, blue vs. green columns). The concentration was selected according to venetoclax human PK studies^4,38^. CFSE profiles of the residual live cells, however, revealed an interesting pattern of distribution (Fig. 3A, middle and right panels), with 97% of dividing cells and 3% of resting cells (green trace). This is in contrast to the respective 66 and 34% in the DMSO control, and 8.7 and 91% in ibrutinib-treated cells. Similar observations were made in another ibrutinib-naive case, UCLL047. Regarding ibrutinib-resistant cases, in UCLL052, venetoclax reduced cell viability from 100 to 37%, and of the remaining live cells, >99% were cells in proliferation and <1% were resting (Fig. 3B, green trace). A similar pattern was also noted in UCLL034, another ibrutinib-resistant case. These data suggest that venetoclax induces cell death, but it preferentially kills the resting CLL subpopulation, which is opposite to the action of ibrutinib.

### Combination of ibrutinib and venetoclax effectively eliminates both resting and dividing CLL subpopulations

**Fig. 4:**
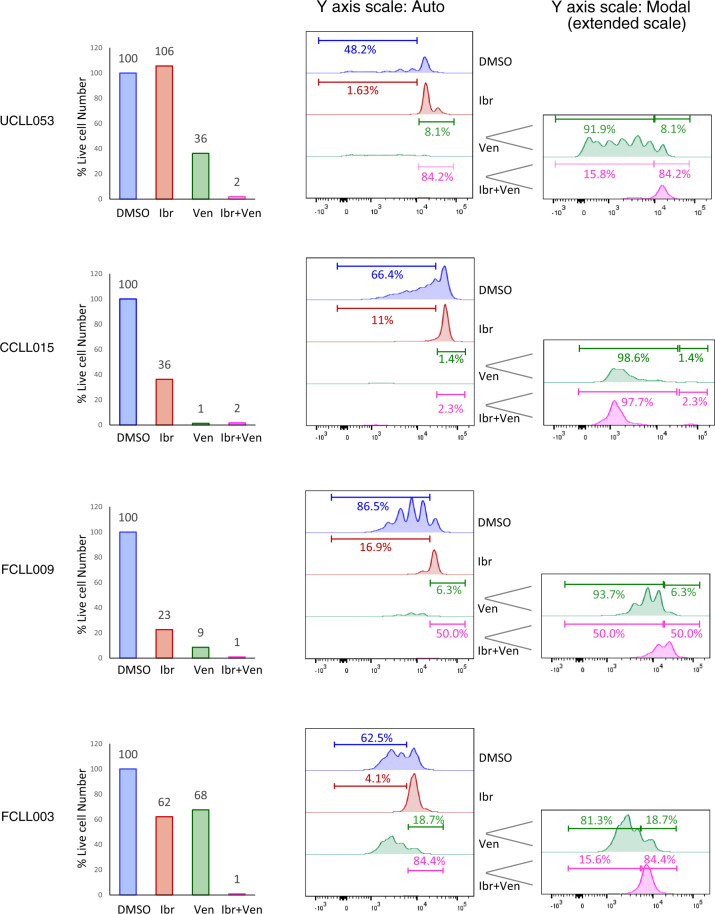
Combination of ibrutinib and venetoclax effectively eliminates both resting and dividing CLL subpopulations in ibrutinib-sensitive cases. CFSE-labeled CLL cells were cultured with BMF+CpG/IL-15 in the presence of DMSO, 400 nM of ibr, 200 nM of venetoclax (ven) or both. All analyses shown were conducted at day 7 of the co-culture by flow cytometry. Live cell numbers (bar graphs) and CFSE profiles of four ibrutinib naïve/sensitive cases are shown. Live cell numbers were normalize to the DMSO control (100%). Percentage of cells distributed in the dividing phases of the CFSE profiles is automatically calculated by the FlowJo software. *Y* axis scale-auto displays events on the same scale for all treatment conditions, and *Y* axis scale-Modal displays events on an extended scale to show low peaks under the conditions of ventoclax or combination treatment.

**Fig. 5:**
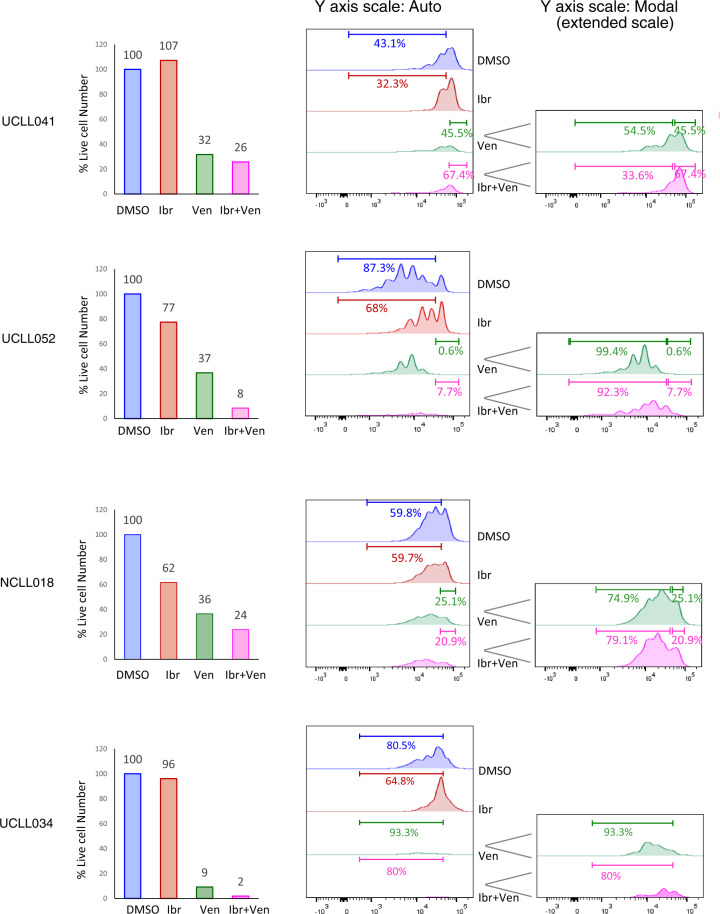
Combination of ibrutinib and venetoclax effectively eliminates both resting and dividing CLL subpopulations in ibrutinib-resistant cases. Live cell numbers and CFSE profiles of four ibrutinib-resistant cases are shown. Experimental conditions were identical to Fig. 4.

With these initial findings that ibrutinib and venetoclax may preferentially act on distinct subpopulations of CLL, we tested the effects of either single drug alone or ibrutinib/venetoclax combination side-by-side in all collected cases. Regarding cell viability, Fig. 4 shows several ibrutinib-naive cases while Fig. 5 demonstrates several ibrutinib-resistant cases. Again, ibrutinib showed a highly variable effect on the number of live cells (Figs. 4 and 5, red columns). Venetoclax, on the other hand, reduced the total number of live cells in every single case evaluated (Figs. 4 and 5, green columns, 1–68% relative to DMSO), irrespective of their sensitivity to ibrutinib, which is consistent with its mechanism of action as a BCL2 inhibitor.

As to cell proliferation, with additional cases evaluated, it became evident that ibrutinib reduced the number of dividing cells in sensitive cases (Fig. 4, blue vs. red traces), 48.2–1.63% in UCLL053, 66.4–11.0% in CCLL015, 86.5–16.9% in FCLL009 and 62.5–4.1% in FCLL003. In comparison, this inhibition was not as effective in the ibrutinib-resistant cases (Fig. 5, blue vs. red traces), 43.1–32.3% in UCLL041, 87.3–68.0% in UCLL052, 59.8–59.7% in NCLL018 and 80.5–64.8% in UCLL034. With regards to venetoclax, notably, the resting CLL cells, as opposed to dividing cells, are the subpopulation mainly targeted by venetoclax. The proportion of the resting cells ranged from 0.6% in UCLL052 to 18.7% in FCLL003 while the dividing cells made up 81.3–99.4% of the total cell populations (Figs. 4 and 5, green traces).

The combination of the two drugs, as expected, worked most effectively, significantly reducing total number of live cells (1–26%) in the vast majority of cases irrespective of ibrutinib-sensitivity (Figs. 4 and 5, pink columns). Analyses of CFSE profiles became less meaningful in many of these cases due to the small number of residual live cells left after the combined treatment.

**Fig. 6:**
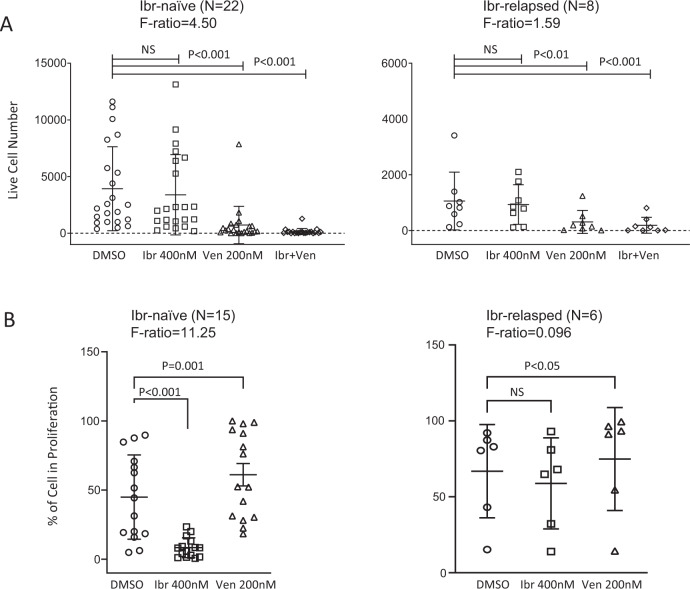
Effects of ibrutinib, venetoclax or combination on cell viability and cell proliferation. **A** Aggregate results of cell viability for ibr naïve patients (left) and ibr resistant patients (right). **B** Aggregate results of percentage of the dividing CLL cells for ibr naïve patients (left) and ibrutinib-resistant patients (right). The combination was not depicted since analyses of the CFSE profiles became less meaningful in many cases due to the small number of residual live cells left after the combined treatment. *p* values and *F* ratios are indicated.

Figure 6A shows the aggregate cell viability data on 22 ibrutinib-naïve and eight ibrutinib-resistant cases. It is apparent that ibrutinib did not exert any significant effects on cell viability (*p* value, not significant), Venetoclax, on the other hand, markedly decreased the number of viable cells in both ibrutinib-naïve (left, *p* < 0.001) and ibrutinib-resistant cases (right, *p* < 0.01). The combination of ibrutinib and venetoclax, thus far, produced the most pronounced cell killing (*p* < 0.001 in both cohorts).

Aggregate cell proliferation data are shown on 15 ibrutinib naïve and six ibrutinib-resistant cases (Fig. 6B). Such analyses for cases with few live cells (<100 events) after venetoclax treatment were excluded since they may not be statistically meaningful. It is clearly evident that ibrutinib significantly reduced the percentage of proliferating CLL cells in sensitive but not resistant cases (Fig. 6B, left vs. right panel *p* < 0.001 vs. ns). Venetoclax, on the other hand, increased the proportion of the proliferating cells (i.e., decreased the proportion of the resting cells) among the residual live cells in both ibrutinib-naïve and ibrutinib-relapsed cases (Fig. 6B, left and right, *p* = 0.001 and *p* < 0.05 respectively).

To further validate these findings, we performed additional analyses of Ki67 expression using flow cytometry. Ki67 is one of the most established and widely used biomarkers for cell proliferation. Compared to CFSE analysis, Ki67 is an earlier marker of cell proliferation and flow cytometric analysis of its expression displays a continuous cell population rather than distinct populations of resting vs. dividing cells. Despite this, the results show that percentages of Ki67^+^ cells were increased by CpG/IL-15 stimulation, and reduced by ibrutinib but not by venetoclax treatment (Fig. S1). These data corroborate our conclusion derived from the CFSE data.

Taken together, the side-by-side comparisons by CFSE staining, for the first time, demonstrate that ibrutinib and venetoclax target *distinct* subpopulations of CLL cells with different proliferative capacities. The data imply that the inability of each of the drugs to act on the entire CLL population may account for the persistence of residual disease. These data collectively provide a strong laboratory rationale that combining these two drugs may have the potential for a cure, consistent with the current clinical observations (see “Discussion” section below).

## Discussion

In summary, using an ex vivo model that promotes CLL cell proliferation, we demonstrated that ibrutinib primarily targets CLL proliferation/division as opposed to inducing cell death, while venetoclax primarily induces cell death, but preferentially the death of resting cells. Treatment of tumor cells with either drug left behind a subpopulation of CLL cells which is indifferent to the monotherapy while the combination essentially eliminates the vast majority of tumor cells. Regarding cell viability, in some cases we observed an unexpected increase after ibrutinib treatment relative to the DMSO control (Fig. 2A). This might be explained by the known inhibitory effect of ibrutinib on cell adhesion^39,40^. Upon treatment with ibrutinib, the live, proliferative CLL cells may become detached from the BMF and released into the media.

The novelty of the work lies on the new model that induces CLL proliferation (Model construction detail will be described separately). Although cell proliferation is not at all a new concept for tumors in general, for CLL, an indolent disease, this property was rarely measured before due to the lack of suitable models. CLL is not merely an indolent disease, tumor cells indeed form “proliferation center” in the lymph nodes. Equipped with the new proliferation assay, we were able to separate cells into dividing and resting subpopulations and see how particular drugs act on subpopulations that has never been demonstrated previously.

Previous studies have provided some laboratory rationale for ibrutinib and venetoclax combination^41,42^. Cervantes-Gomez and colleagues performed ex vivo and in vitro studies on residual circulating CLL cells from patients on ibrutinib treatment. They found that venetoclax induced a significant degree of cell death, and concluded that venetoclax is an optimal partner with ibrutinib^41^. However, the PB CLL cells were treated as a whole cell population in this study without stromal support. Deng et al. have found that BTK inhibition enhanced mitochondrial BCL2 dependence and pretreatment of CLL cells with BTK inhibitors enhanced the killing by venetoclax^42^. These independent studies support the strategy of combining BTK and BCL2 inhibitors as well as ours. However, our study advanced the current knowledge by demonstrating that CLL subpopulations with different proliferative capacities have different sensitivity to ibrutinib and venetoclax, respectively. The effects on cellular subpopulations have never been seen before due to the limitation of the prior experimental systems.

Overall, our data are consistent with a number of clinical observations. It is learned that ibrutinib is more effective against nodal disease with rapid reduction of lymphadenopathy, while venetoclax is more effective against PB disease^4,43^. Previously, using gene expression microarray, Herishanu and colleagues demonstrated that CLL cells residing in different anatomic quarters have different proliferative capacity. LN CLL cells, in particular, are more proliferative than the PB CLL cells, as evidenced by a higher expression of E2F-target and MYC-target genes and a higher fraction of Ki-67^+^ cells^44^. Putting this together with our laboratory data, the compartmental responses to the BTKi and BCL2i can now be explained by the different proliferative capacities of the CLL subpopulations residing in these different anatomical compartments.

In addition, our results may contribute to the understanding of several other clinical observations. (1) The complete response rate of ibrutinib monotherapy remains low at 10% for R/R patients and <30% for treatment-naïve patients after a 5-year follow-up^45^. According to our current and previous findings^25–27,32^, this results from little direct cell killing rendered by ibrutinib. On the contrary, tumor lysis syndrome associated with venetoclax can be explained by the direct killing of the resting CLL cells in the periphery. (2) Complete remission with uMRD has been low for ibrutinib or venetoclax monotherapy, especially ibrutinib alone. In contrast, the combination has produced durable responses with high rates of complete remission and uMRD which continuously increases over time^7,8^. This observation was reproduced in our ex vivo system where the BTK and BCL2 inhibitors target distinct subpopulations of CLL cells. Together they leave few tumor cells unscathed.

Besides proliferation, ibrutinib also plays a key role in disrupting cell adhesion^39,40,46^. Clinically, when ibrutinib is started, it is widely observed that a rapid reduction in lymphadenopathy is accompanied by a marked increase in circulating lymphocytes. Disruption of tumor cell adherence to nodal stroma is attributed as the cause of this egress. Inhibition of cell proliferation and disruption of cell adhesion are believed to happen in parallel with ibrutinib treatment.

**Fig. 7:**
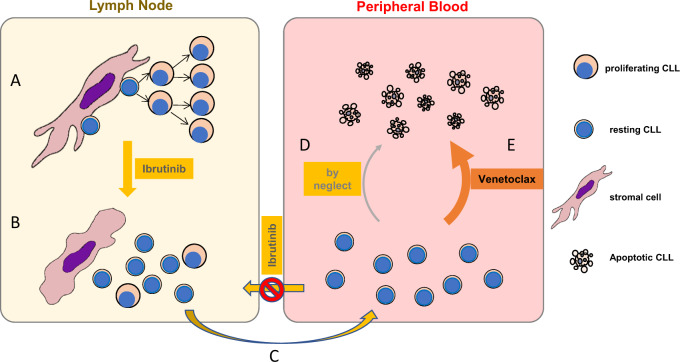
A schematic diagram of tumor cell dynamics under ibrutinib or venetoclax treatment. **A** In lymph nodes, with close contact between tumor and stroma, CLL cells are well supported and proliferate. **B** With ibrutinibutinib treatment, adhesion to stromal cells is disrupted and proliferation is halted. **C** CLL cells are released to the periphery where they are also prevented from homing back to LN by the action of ibrutinibutinib on chemokine signaling. **D** In the periphery, without the supporting tumor microenvironment, the cells become non-proliferating/resting and die by neglect over a long period of time. **E** In the presence of venetoclax, however, these resting cells are subjective to active killing, causing tumor lysis syndrome and disease improvement.

Integrating clinical and laboratory data, the following tumor cell dynamic can be envisioned when patients are treated with either single drug alone or the drug combination (Fig. 7): In LN, with close contact between tumor and stroma, CLL cells are well supported and proliferate; with ibrutinib treatment, adhesion to stromal cells is disrupted and proliferation is halted. Subsequently, CLL cells are released to the periphery where they are also prevented from homing back to LN by the action of ibrutinib on chemokine signaling. In the periphery, without the supporting tumor microenvironment, the cells become non-proliferating/resting and die by neglect over a relatively long period of time; in the presence of venetoclax, however, these resting cells are subjected to active killing, causing tumor lysis syndrome and tumor burden improvement. This proposed dynamic is compatible with current laboratory data and clinical experience but may need further investigational proof.

Altogether, our study suggests that the combination, with its ability to target both subpopulations of tumor cells, has the greatest potential to minimize or even eradicate MRD at all anatomic sites, which would eventually translate to a reduction in future relapses. In addition, our model or other similar models, may become a useful and practical tool for testing the effectiveness of other drugs or drug combinations, not only on cell viability but also on cell proliferation. Cell adhesion, another critical cellular behavior, may also be evaluated using these models.

## Supplementary information

Supplemental Figure 1

## References

[1] Rozman C, Montserrat E (1995). Chronic lymphocytic leukemia. N. Engl. J. Med..

[2] Chiorazzi N, Rai KR, Ferrarini M (2005). Chronic lymphocytic leukemia. N. Engl. J. Med..

[3] Byrd JC (2013). Targeting BTK with ibrutinib in relapsed chronic lymphocytic leukemia. N. Engl. J. Med..

[4] Roberts AW (2016). Targeting BCL2 with venetoclax in relapsed chronic lymphocytic leukemia. N. Engl. J. Med..

[5] Roberts AW (2019). Efficacy of venetoclax in relapsed chronic lymphocytic leukemia is influenced by disease and response variables. Blood.

[6] Lee J, Wang YL (2020). Prognostic and predictive molecular biomarkers in chronic lymphocytic leukemia. J. Mol. Diagn..

[7] Hillmen P (2019). Ibrutinib plus venetoclax in relapsed/refractory chronic lymphocytic leukemia: the CLARITY Study. J. Clin. Oncol..

[8] Jain N (2019). Ibrutinib and venetoclax for first-line treatment of CLL. N. Engl. J. Med..

[9] Deaglio S, Malavasi F (2009). Chronic lymphocytic leukemia microenvironment: shifting the balance from apoptosis to proliferation. Haematologica.

[10] Messmer BT (2005). In vivo measurements document the dynamic cellular kinetics of chronic lymphocytic leukemia B cells. J. Clin. Investig..

[11] Schmid C, Isaacson PG (1994). Proliferation centres in B-cell malignant lymphoma, lymphocytic (B-CLL): an immunophenotypic study. Histopathology.

[12] Lampert IA, Wotherspoon A, Van, Noorden S, Hasserjian RP (1999). High expression of CD23 in the proliferation centers of chronic lymphocytic leukemia in lymph nodes and spleen. Hum. Pathol..

[13] Fabbri G, Dalla-Favera R (2016). The molecular pathogenesis of chronic lymphocytic leukaemia. Nat. Rev. Cancer.

[14] Gine E (2010). Expanded and highly active proliferation centers identify a histological subtype of chronic lymphocytic leukemia (“accelerated” chronic lymphocytic leukemia) with aggressive clinical behavior. Haematologica.

[15] Song Z (2010). Activities of SYK and PLCgamma2 predict apoptotic response of CLL cells to SRC tyrosine kinase inhibitor dasatinib. Clin. Cancer Res..

[16] Herman SE (2011). Bruton tyrosine kinase represents a promising therapeutic target for treatment of chronic lymphocytic leukemia and is effectively targeted by PCI-32765. Blood.

[17] Herishanu Y, Katz BZ, Lipsky A, Wiestner A (2013). Biology of chronic lymphocytic leukemia in different microenvironments: clinical and therapeutic implications. Hematol. Oncol. Clin. N. Am..

[18] Caligaris-Cappio F, Bertilaccio MT, Scielzo C (2014). How the microenvironment wires the natural history of chronic lymphocytic leukemia. Semin. Cancer Biol..

[19] Oppezzo P, Dighiero G (2013). Role of the B-cell receptor and the microenvironment in chronic lymphocytic leukemia. Blood Cancer J..

[20] Kurtova AV (2009). Diverse marrow stromal cells protect CLL cells from spontaneous and drug-induced apoptosis: development of a reliable and reproducible system to assess stromal cell adhesion-mediated drug resistance. Blood.

[21] Janel A (2014). The chronic lymphocytic leukemia clone disrupts the bone marrow microenvironment. Stem Cells Dev..

[22] Purroy N (2015). Co-culture of primary CLL cells with bone marrow mesenchymal cells, CD40 ligand and CpG ODN promotes proliferation of chemoresistant CLL cells phenotypically comparable to those proliferating in vivo. Oncotarget.

[23] Bottcher S (2012). Minimal residual disease quantification is an independent predictor of progression-free and overall survival in chronic lymphocytic leukemia: a multivariate analysis from the randomized GCLLSG CLL8 trial. J. Clin. Oncol..

[24] Bai C (2015). Multi-lineage potential research of bone marrow mesenchymal stem cells from Bama miniature pig. J. Exp. Zool. Part B.

[25] Cheng S (2015). Functional characterization of BTK(C481S) mutation that confers ibrutinib resistance: exploration of alternative kinase inhibitors. Leukemia.

[26] Guo A (2016). Heightened BTK-dependent cell proliferation in unmutated chronic lymphocytic leukemia confers increased sensitivity to ibrutinib. Oncotarget.

[27] Guo A (2017). Dual SYK/JAK inhibition overcomes ibrutinib resistance in chronic lymphocytic leukemia: Cerdulatinib, but not ibrutinib, induces apoptosis of tumor cells protected by the microenvironment. Oncotarget.

[28] Guo A (2017). HSP90 stabilizes B-cell receptor kinases in a multi-client interactome: PU-H71 induces CLL apoptosis in a cytoprotective microenvironment. Oncogene.

[29] Mongini PK (2015). TLR-9 and IL-15 synergy promotes the in vitro clonal expansion of chronic lymphocytic leukemia B cells. J. Immunol..

[30] Gupta R (2018). Mechanistic insights into CpG DNA and IL-15 synergy in promoting B cell chronic lymphocytic leukemia clonal expansion. J. Immunol..

[31] Calissano C (2011). Intraclonal complexity in chronic lymphocytic leukemia: fractions enriched in recently born/divided and older/quiescent cells. Mol. Med..

[32] Cheng S (2014). BTK inhibition targets in vivo CLL proliferation through its effects on B-cell receptor signaling activity. Leukemia.

[33] Advani RH (2013). Bruton tyrosine kinase inhibitor ibrutinib (PCI-32765) has significant activity in patients with relapsed/refractory B-cell malignancies. J. Clin. Oncol..

[34] Ma J (2014). Characterization of ibrutinib-sensitive and -resistant mantle lymphoma cells. Br. J. Haematol..

[35] Ming M (2018). XPO1 inhibitor selinexor overcomes intrinsic ibrutinib resistance in mantle cell lymphoma via nuclear retention of IkappaB. Mol. Cancer Ther..

[36] Furman RR (2014). Ibrutinib resistance in chronic lymphocytic leukemia. N. Engl. J. Med..

[37] Burger JA (2017). Leukemia cell proliferation and death in chronic lymphocytic leukemia patients on therapy with the BTK inhibitor ibrutinib. JCI Insight.

[38] Liu H (2017). Metabolism and disposition of a novel B-cell lymphoma-2 inhibitor venetoclax in humans and characterization of its unusual metabolites. Drug Metabol. Dispos..

[39] de Rooij MF (2012). The clinically active BTK inhibitor PCI-32765 targets B-cell receptor- and chemokine-controlled adhesion and migration in chronic lymphocytic leukemia. Blood.

[40] Herman SE (2015). Treatment with ibrutinib inhibits BTK- and VLA-4-dependent adhesion of chronic lymphocytic leukemia cells in vivo. Clin. Cancer Res..

[41] Cervantes-Gomez F (2015). Pharmacological and protein profiling suggests venetoclax (ABT-199) as optimal partner with ibrutinib in chronic lymphocytic leukemia. Clin. Cancer Res..

[42] Deng J (2017). Bruton’s tyrosine kinase inhibition increases BCL-2 dependence and enhances sensitivity to venetoclax in chronic lymphocytic leukemia. Leukemia.

[43] Woyach JA (2014). Prolonged lymphocytosis during ibrutinib therapy is associated with distinct molecular characteristics and does not indicate a suboptimal response to therapy. Blood.

[44] Herishanu Y (2011). The lymph node microenvironment promotes B-cell receptor signaling, NF-kappaB activation, and tumor proliferation in chronic lymphocytic leukemia. Blood.

[45] O’Brien S (2018). Single-agent ibrutinib in treatment-naive and relapsed/refractory chronic lymphocytic leukemia: a 5-year experience. Blood.

[46] Kashyap MK (2017). Targeting the CXCR4 pathway using a novel anti-CXCR4 IgG1 antibody (PF-06747143) in chronic lymphocytic leukemia. J. Hematol. Oncol..

