# Supplementary figures and images for: Ibrutinib and venetoclax target distinct subpopulations of CLL cells: implication for residual disease eradication
Source: Blood Cancer J. 2021 Feb 18;11(2):39. doi: 10.1038/s41408-021-00429-z (PMC7893066; doi:10.1038/s41408-021-00429-z)

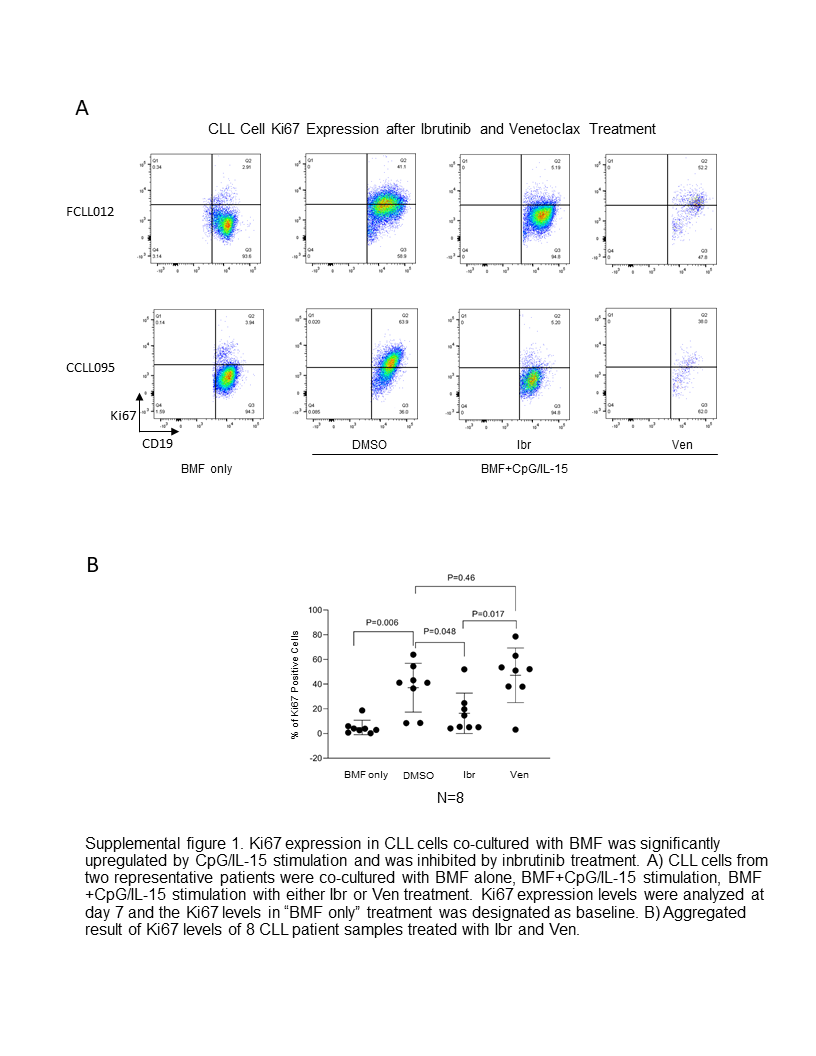

Supplement: Supplementary file 1 — Supplemental Figure 1 [file 41408_2021_429_MOESM1_ESM.tif]
